# Azido{4,4′-dibromo-2,2′-[ethane-1,2-diyl­bis(nitrilo­methanylylidene)]diphenol­ato-κ^4^ *O*,*N*,*N*′,*O*′}manganese(III)

**DOI:** 10.1107/S1600536811004594

**Published:** 2011-02-12

**Authors:** Yingxia Liu

**Affiliations:** a School of Environmental and Material Engineering, Yantai University, Yantai 264005, People’s Republic of China

## Abstract

In the title compound, [Mn(C_16_H_12_Br_2_N_2_O_2_)(N_3_)], the Mn^III^ ion is chelated by a tetra­dentate Schiff base ligand and coordinated by the N atom of an azide ligand in a distorted square-pyramidal arrangement. It forms phenolate-bridged out-of-plane dimers with Mn⋯O_phenolate_ distances of 2.667 (2) Å between pairs of inversion-related mol­ecules. In the crystal, there are offset inter-complex face-to-face π–π inter­actions [centroid–centroid distances = 3.598 (2) Å] involving one of the benzene rings of the ligands.

## Related literature

For related structures, see: Mikuriya *et al.* (1992 ▶); Li *et al.* (1997 ▶); Lu *et al.* (2006 ▶); Wang *et al.* (2008 ▶).
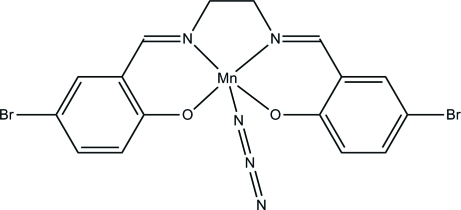


## Experimental

### 

#### Crystal data

[Mn(C_16_H_12_Br_2_N_2_O_2_)(N_3_)]
*M* *_r_* = 1042.13
Monoclinic, 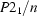

*a* = 8.7068 (17) Å
*b* = 15.269 (3) Å
*c* = 13.684 (3) Å
β = 107.47 (3)°
*V* = 1735.4 (6) Å^3^
*Z* = 2
Mo *K*α radiation
μ = 5.39 mm^−1^
*T* = 153 K
0.20 × 0.17 × 0.10 mm

#### Data collection

Nonius KappaCCD diffractometer
Absorption correction: multi-scan (*SORTAV*; Blessing, 1995 ▶) *T*_min_ = 0.352, *T*_max_ = 0.583
7747 measured reflections
3961 independent reflections
3093 reflections with *I* > 2σ(*I*)
*R*_int_ = 0.015

#### Refinement

*R*[*F* ^2^ > 2σ(*F* ^2^)] = 0.030
*wR*(*F* ^2^) = 0.074
*S* = 1.04
3961 reflections
235 parameters
H-atom parameters constrained
Δρ_max_ = 0.82 e Å^−3^
Δρ_min_ = −0.90 e Å^−3^

### 

Data collection: *COLLECT* (Nonius, 1998 ▶); cell refinement: *SCALEPACK* (Otwinowski & Minor, 1997 ▶); data reduction: *DENZO* (Otwinowski & Minor, 1997 ▶) and *maXus* (Mackay *et al.*, 1998 ▶); program(s) used to solve structure: *SHELXS97* (Sheldrick, 2008 ▶); program(s) used to refine structure: *SHELXL97* (Sheldrick, 2008 ▶); molecular graphics: *SHELXTL* (Sheldrick, 2008 ▶); software used to prepare material for publication: *SHELXL97*.

## Supplementary Material

Crystal structure: contains datablocks I, global. DOI: 10.1107/S1600536811004594/pk2299sup1.cif

Structure factors: contains datablocks I. DOI: 10.1107/S1600536811004594/pk2299Isup2.hkl

Additional supplementary materials:  crystallographic information; 3D view; checkCIF report

## Figures and Tables

**Fig. 1.**
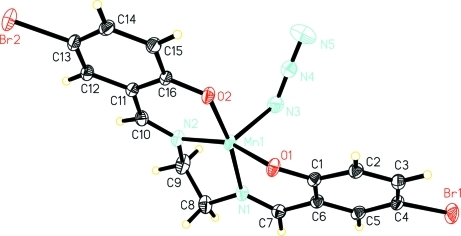
The molecular structure (30% thermal probability ellipsoids) of the compound showing the atom numbering.

**Fig. 2.**
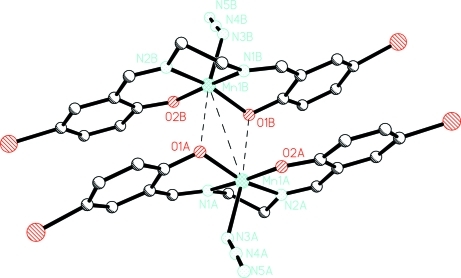
A pair of inversion-related molecules, showing the intermolecular weak interactions between Mn and Ophenolate atoms. The 'A' molecule is related to the 'B' molecule by [A: (x, y, z) —> B: (2-x, 1-y, -z)].

## supplementary crystallographic information

### Comment

In the past decade there has been much interest in the magneto-chemistry of manganese because of its special magnetic properties. As is well known, manganese (III) Schiff base complexes display interesting structural, magnetic properties and electronic effects which rank it among the most appealing candidates as a building paramagnetic motif for multidimensional expanded structures. The variation of in-plane chelating and axial sites often leads to a change in the spin state of the metal ions: high-spin, low-spin or spin-crossover state. The nature and the tuning of magnetic interactions between metal centers are crucial points in the conception of molecular-based magnetic materials.

The molecular structure of the title compound is shown in Figure 1. The Mn^III^ ion is involved in a distorted square-pyramidal arrangement by a N3O2 unit, in which the four basal sites are occupied by two N atoms and two O atoms from the Schiff base ligand, and the apical position is occupied by the N atom of an azido ligand. The bond distances are comparable to those found in related structures (Lu, *et al.*, 2006; Mikuriya, *et al.*, 1992; Li, *et al.*, 1997; Wang, *et al.*, 2008). The Mn^III^ ion lies above the basal plane formed by N2O2 unit by 0.228 (1) Å. The short intermolecular distance of Mn···O_phenolate_ 2.667 (2) Å indicates that there exists weak interaction between the two complexes related by inversion centers in the crystal (Figure 2). The phenyl groups of the Schiff base are involved in an offset face-to-face π-π inter-complexes stacking interaction (ring centroid separation *C*g···*C*g^i^, 3.598 (2) Å) [symmetry code: 2 - *x*,1 - *y*,1 - *z*].

### Experimental

This compound was synthesized by mixing a solution of Schiff base (2,2'-((1*E*,1'*E*)-(ethane-1,2-diylbis(azanylylidene)) bis(methanylylidene))bis(4-bromophenol)) (0.5 mmol) in methanol (5 ml) with a solution of MnCl_2_.4H_2_O (0.5 mmol) in methanol (5 ml), followed by the dropwise addition of an aqueous solution NaN_3_(0.6 mmol, 2 mL) without stirring. The black mixture was allowed to stand for several days until good quality black block crystals of the compound were obtained in a yield of 68.3%.

### Refinement

All the H atoms bonded to the C atoms were placed using the HFIX commands in *SHELXL97* (Sheldrick, 2008) with C—H distances of 0.93 and 0.97 Å, respectively, and were allowed for as riding atoms with *U*_iso_(H) = 1.2Ueq(C).

### Crystal data

**Table:**

[Mn(C_16_H_12_Br_2_N_2_O_2_)(N_3_)]	*F*(000) = 1016
*M**_r_* = 1042.13	*D*_x_ = 1.994 Mg m^−^^3^
Monoclinic, *P*2_1_/*n*	Mo *K*α radiation, λ = 0.71073 Å
Hall symbol: -P 2yn	Cell parameters from 19417 reflections
*a* = 8.7068 (17) Å	θ = 3.4–27.5°
*b* = 15.269 (3) Å	µ = 5.39 mm^−^^1^
*c* = 13.684 (3) Å	*T* = 153 K
β = 107.47 (3)°	Block, black
*V* = 1735.4 (6) Å^3^	0.20 × 0.17 × 0.10 mm
*Z* = 2	

### Data collection

**Table:**

Nonius KappaCCD diffractometer	3961 independent reflections
Radiation source: fine-focus sealed tube	3093 reflections with *I* > 2σ(*I*)
graphite	*R*_int_ = 0.015
ω scans	θ_max_ = 27.5°, θ_min_ = 3.5°
Absorption correction: multi-scan (*SORTAV*; Blessing, 1995)	*h* = −11→11
*T*_min_ = 0.352, *T*_max_ = 0.583	*k* = −19→19
7747 measured reflections	*l* = −17→17

### Refinement

**Table:**

Refinement on *F*^2^	Primary atom site location: structure-invariant direct methods
Least-squares matrix: full	Secondary atom site location: difference Fourier map
*R*[*F*^2^ > 2σ(*F*^2^)] = 0.030	Hydrogen site location: inferred from neighbouring sites
*wR*(*F*^2^) = 0.074	H-atom parameters constrained
*S* = 1.04	*w* = 1/[σ^2^(*F*_o_^2^) + (0.0376*P*)^2^ + 0.8161*P*] where *P* = (*F*_o_^2^ + 2*F*_c_^2^)/3
3961 reflections	(Δ/σ)_max_ = 0.001
235 parameters	Δρ_max_ = 0.82 e Å^−^^3^
0 restraints	Δρ_min_ = −0.90 e Å^−^^3^

### Special details

**Table:**

Geometry. All e.s.d.'s (except the e.s.d. in the dihedral angle between two l.s. planes) are estimated using the full covariance matrix. The cell e.s.d.'s are taken into account individually in the estimation of e.s.d.'s in distances, angles and torsion angles; correlations between e.s.d.'s in cell parameters are only used when they are defined by crystal symmetry. An approximate (isotropic) treatment of cell e.s.d.'s is used for estimating e.s.d.'s involving l.s. planes.
Refinement. Refinement of *F*^2^ against ALL reflections. The weighted *R*-factor *wR* and goodness of fit *S* are based on *F*^2^, conventional *R*-factors *R* are based on *F*, with *F* set to zero for negative *F*^2^. The threshold expression of *F*^2^ > σ(*F*^2^) is used only for calculating *R*-factors(gt) *etc*. and is not relevant to the choice of reflections for refinement. *R*-factors based on *F*^2^ are statistically about twice as large as those based on *F*, and *R*- factors based on ALL data will be even larger.

### Fractional atomic coordinates and isotropic or equivalent isotropic displacement parameters (Å^2^)

**Table:**

	*x*	*y*	*z*	*U*_iso_*/*U*_eq_	
Mn1	0.85052 (5)	0.50918 (2)	0.38001 (3)	0.02912 (10)	
Br1	1.11255 (4)	0.95137 (2)	0.37896 (3)	0.05433 (11)	
Br2	0.43251 (4)	0.106181 (19)	0.44904 (2)	0.04785 (10)	
C1	0.9787 (3)	0.67065 (17)	0.46365 (19)	0.0282 (5)	
C2	0.9438 (3)	0.74469 (17)	0.5129 (2)	0.0323 (6)	
H2	0.8919	0.7381	0.5627	0.039*	
C3	0.9851 (3)	0.82750 (18)	0.4890 (2)	0.0358 (6)	
H3	0.9580	0.8764	0.5208	0.043*	
C4	1.0675 (3)	0.83713 (17)	0.4168 (2)	0.0336 (6)	
C5	1.1113 (3)	0.76578 (18)	0.3710 (2)	0.0321 (6)	
H5	1.1715	0.7732	0.3258	0.039*	
C6	1.0655 (3)	0.68135 (17)	0.39187 (19)	0.0279 (5)	
C7	1.1100 (3)	0.60754 (17)	0.33963 (19)	0.0307 (5)	
H7	1.1928	0.6147	0.3104	0.037*	
C8	1.0902 (4)	0.45714 (18)	0.2823 (2)	0.0403 (7)	
H8A	1.1603	0.4191	0.3333	0.048*	
H8B	1.1476	0.4763	0.2354	0.048*	
C9	0.9380 (4)	0.4093 (2)	0.2250 (2)	0.0446 (7)	
H9A	0.8813	0.4411	0.1634	0.054*	
H9B	0.9636	0.3512	0.2056	0.054*	
C10	0.7619 (3)	0.33187 (17)	0.30160 (19)	0.0314 (5)	
H10	0.7730	0.2845	0.2615	0.038*	
C11	0.6617 (3)	0.32053 (16)	0.36746 (19)	0.0274 (5)	
C12	0.6028 (3)	0.23581 (17)	0.37468 (19)	0.0309 (5)	
H12	0.6289	0.1898	0.3380	0.037*	
C13	0.5066 (3)	0.22139 (16)	0.4361 (2)	0.0316 (6)	
C14	0.4649 (3)	0.28895 (18)	0.4908 (2)	0.0348 (6)	
H14	0.3986	0.2780	0.5315	0.042*	
C15	0.5215 (3)	0.37191 (18)	0.4848 (2)	0.0344 (6)	
H15	0.4912	0.4173	0.5204	0.041*	
C16	0.6248 (3)	0.38931 (16)	0.4256 (2)	0.0285 (5)	
N1	1.0407 (3)	0.53307 (14)	0.33190 (16)	0.0304 (5)	
N2	0.8372 (3)	0.40293 (14)	0.29446 (16)	0.0314 (5)	
N3	0.7109 (3)	0.60055 (17)	0.2717 (2)	0.0449 (6)	
N4	0.5810 (3)	0.62722 (17)	0.26329 (18)	0.0412 (6)	
N5	0.4533 (4)	0.6552 (3)	0.2518 (3)	0.0747 (10)	
O1	0.9342 (2)	0.59151 (11)	0.48779 (14)	0.0333 (4)	
O2	0.6852 (2)	0.46910 (12)	0.42930 (15)	0.0362 (4)	

### Atomic displacement parameters (Å^2^)

**Table:**

	*U*^11^	*U*^22^	*U*^33^	*U*^12^	*U*^13^	*U*^23^
Mn1	0.0366 (2)	0.02311 (19)	0.0337 (2)	−0.00517 (16)	0.01976 (18)	−0.00327 (16)
Br1	0.0655 (2)	0.02778 (16)	0.0791 (3)	−0.00838 (14)	0.03595 (19)	0.00547 (15)
Br2	0.0672 (2)	0.02952 (16)	0.05173 (19)	−0.01626 (14)	0.02536 (16)	−0.00135 (13)
C1	0.0296 (12)	0.0273 (13)	0.0273 (12)	−0.0038 (10)	0.0079 (11)	0.0013 (10)
C2	0.0329 (13)	0.0305 (13)	0.0362 (14)	−0.0032 (11)	0.0147 (12)	−0.0028 (11)
C3	0.0336 (14)	0.0287 (14)	0.0472 (16)	−0.0010 (11)	0.0154 (13)	−0.0036 (12)
C4	0.0332 (14)	0.0243 (12)	0.0417 (15)	−0.0062 (11)	0.0090 (12)	0.0028 (11)
C5	0.0304 (13)	0.0330 (14)	0.0331 (14)	−0.0066 (11)	0.0096 (11)	0.0033 (11)
C6	0.0284 (12)	0.0273 (13)	0.0285 (13)	−0.0026 (10)	0.0092 (11)	0.0025 (10)
C7	0.0322 (13)	0.0331 (14)	0.0293 (13)	−0.0012 (11)	0.0129 (11)	0.0040 (11)
C8	0.0549 (18)	0.0305 (14)	0.0490 (17)	0.0016 (13)	0.0359 (15)	−0.0012 (12)
C9	0.070 (2)	0.0367 (16)	0.0416 (16)	−0.0095 (14)	0.0383 (16)	−0.0092 (13)
C10	0.0389 (14)	0.0269 (13)	0.0282 (13)	0.0003 (11)	0.0101 (11)	−0.0034 (10)
C11	0.0288 (12)	0.0250 (13)	0.0282 (13)	−0.0032 (10)	0.0080 (11)	−0.0008 (10)
C12	0.0363 (14)	0.0244 (13)	0.0305 (13)	−0.0038 (11)	0.0076 (11)	−0.0027 (10)
C13	0.0355 (14)	0.0239 (13)	0.0327 (13)	−0.0065 (10)	0.0061 (12)	0.0009 (10)
C14	0.0326 (14)	0.0335 (14)	0.0417 (15)	−0.0060 (11)	0.0162 (13)	−0.0004 (12)
C15	0.0349 (14)	0.0295 (14)	0.0438 (16)	−0.0020 (11)	0.0194 (13)	−0.0047 (12)
C16	0.0290 (12)	0.0219 (12)	0.0357 (14)	−0.0037 (10)	0.0112 (11)	−0.0011 (10)
N1	0.0386 (12)	0.0272 (11)	0.0309 (11)	−0.0008 (9)	0.0189 (10)	0.0017 (9)
N2	0.0432 (12)	0.0282 (11)	0.0283 (11)	−0.0045 (10)	0.0190 (10)	−0.0033 (9)
N3	0.0442 (15)	0.0419 (15)	0.0490 (15)	−0.0026 (12)	0.0146 (12)	0.0109 (12)
N4	0.0473 (15)	0.0413 (14)	0.0349 (13)	−0.0036 (12)	0.0121 (12)	0.0038 (11)
N5	0.061 (2)	0.105 (3)	0.060 (2)	0.028 (2)	0.0223 (17)	0.0147 (19)
O1	0.0480 (11)	0.0255 (9)	0.0327 (9)	−0.0090 (8)	0.0215 (9)	−0.0016 (7)
O2	0.0427 (11)	0.0239 (9)	0.0514 (12)	−0.0061 (8)	0.0283 (10)	−0.0083 (8)

### Geometric parameters (Å, °)

**Table:**

Mn1—O2	1.8669 (18)	C8—C9	1.511 (4)
Mn1—O1	1.9083 (19)	C8—H8A	0.9700
Mn1—N2	1.984 (2)	C8—H8B	0.9700
Mn1—N1	1.991 (2)	C9—N2	1.478 (3)
Mn1—N3	2.130 (3)	C9—H9A	0.9700
Br1—C4	1.894 (3)	C9—H9B	0.9700
Br2—C13	1.900 (2)	C10—N2	1.286 (3)
C1—O1	1.340 (3)	C10—C11	1.441 (3)
C1—C2	1.396 (4)	C10—H10	0.9300
C1—C6	1.418 (3)	C11—C12	1.406 (3)
C2—C3	1.381 (4)	C11—C16	1.411 (3)
C2—H2	0.9300	C12—C13	1.371 (4)
C3—C4	1.392 (4)	C12—H12	0.9300
C3—H3	0.9300	C13—C14	1.385 (4)
C4—C5	1.367 (4)	C14—C15	1.371 (4)
C5—C6	1.404 (4)	C14—H14	0.9300
C5—H5	0.9300	C15—C16	1.405 (4)
C6—C7	1.448 (4)	C15—H15	0.9300
C7—N1	1.277 (3)	C16—O2	1.322 (3)
C7—H7	0.9300	N3—N4	1.175 (3)
C8—N1	1.472 (3)	N4—N5	1.157 (4)
			
O2—Mn1—O1	95.38 (8)	N2—C9—C8	107.2 (2)
O2—Mn1—N2	91.73 (8)	N2—C9—H9A	110.3
O1—Mn1—N2	159.40 (9)	C8—C9—H9A	110.3
O2—Mn1—N1	171.04 (9)	N2—C9—H9B	110.3
O1—Mn1—N1	88.41 (9)	C8—C9—H9B	110.3
N2—Mn1—N1	82.06 (9)	H9A—C9—H9B	108.5
O2—Mn1—N3	97.19 (10)	N2—C10—C11	124.5 (2)
O1—Mn1—N3	96.43 (10)	N2—C10—H10	117.7
N2—Mn1—N3	101.83 (10)	C11—C10—H10	117.7
N1—Mn1—N3	90.43 (9)	C12—C11—C16	119.7 (2)
O1—C1—C2	119.4 (2)	C12—C11—C10	117.2 (2)
O1—C1—C6	121.9 (2)	C16—C11—C10	123.1 (2)
C2—C1—C6	118.7 (2)	C13—C12—C11	119.6 (2)
C3—C2—C1	121.2 (2)	C13—C12—H12	120.2
C3—C2—H2	119.4	C11—C12—H12	120.2
C1—C2—H2	119.4	C12—C13—C14	121.3 (2)
C2—C3—C4	119.4 (3)	C12—C13—Br2	119.5 (2)
C2—C3—H3	120.3	C14—C13—Br2	119.22 (19)
C4—C3—H3	120.3	C15—C14—C13	119.9 (2)
C5—C4—C3	121.0 (2)	C15—C14—H14	120.0
C5—C4—Br1	119.94 (19)	C13—C14—H14	120.0
C3—C4—Br1	119.0 (2)	C14—C15—C16	120.9 (2)
C4—C5—C6	120.2 (2)	C14—C15—H15	119.6
C4—C5—H5	119.9	C16—C15—H15	119.6
C6—C5—H5	119.9	O2—C16—C15	117.9 (2)
C5—C6—C1	119.3 (2)	O2—C16—C11	123.6 (2)
C5—C6—C7	118.7 (2)	C15—C16—C11	118.5 (2)
C1—C6—C7	122.0 (2)	C7—N1—C8	122.9 (2)
N1—C7—C6	123.0 (2)	C7—N1—Mn1	123.72 (18)
N1—C7—H7	118.5	C8—N1—Mn1	113.33 (16)
C6—C7—H7	118.5	C10—N2—C9	121.2 (2)
N1—C8—C9	106.7 (2)	C10—N2—Mn1	125.88 (17)
N1—C8—H8A	110.4	C9—N2—Mn1	112.66 (17)
C9—C8—H8A	110.4	N4—N3—Mn1	128.8 (2)
N1—C8—H8B	110.4	N5—N4—N3	177.5 (3)
C9—C8—H8B	110.4	C1—O1—Mn1	118.36 (16)
H8A—C8—H8B	108.6	C16—O2—Mn1	128.97 (16)
			
O1—C1—C2—C3	−178.9 (3)	O1—Mn1—N1—C7	−33.1 (2)
C6—C1—C2—C3	3.3 (4)	N2—Mn1—N1—C7	165.2 (2)
C1—C2—C3—C4	−2.1 (4)	N3—Mn1—N1—C7	63.3 (2)
C2—C3—C4—C5	−1.4 (4)	O2—Mn1—N1—C8	34.1 (7)
C2—C3—C4—Br1	176.4 (2)	O1—Mn1—N1—C8	149.32 (19)
C3—C4—C5—C6	3.6 (4)	N2—Mn1—N1—C8	−12.37 (19)
Br1—C4—C5—C6	−174.3 (2)	N3—Mn1—N1—C8	−114.3 (2)
C4—C5—C6—C1	−2.3 (4)	C11—C10—N2—C9	179.9 (3)
C4—C5—C6—C7	178.2 (2)	C11—C10—N2—Mn1	6.0 (4)
O1—C1—C6—C5	−178.9 (2)	C8—C9—N2—C10	−137.6 (3)
C2—C1—C6—C5	−1.2 (4)	C8—C9—N2—Mn1	37.1 (3)
O1—C1—C6—C7	0.6 (4)	O2—Mn1—N2—C10	−13.6 (2)
C2—C1—C6—C7	178.3 (2)	O1—Mn1—N2—C10	96.7 (3)
C5—C6—C7—N1	−161.0 (3)	N1—Mn1—N2—C10	159.9 (2)
C1—C6—C7—N1	19.5 (4)	N3—Mn1—N2—C10	−111.3 (2)
N1—C8—C9—N2	−45.4 (3)	O2—Mn1—N2—C9	172.0 (2)
N2—C10—C11—C12	−173.0 (3)	O1—Mn1—N2—C9	−77.7 (3)
N2—C10—C11—C16	5.3 (4)	N1—Mn1—N2—C9	−14.4 (2)
C16—C11—C12—C13	1.7 (4)	N3—Mn1—N2—C9	74.3 (2)
C10—C11—C12—C13	−179.9 (2)	O2—Mn1—N3—N4	12.9 (3)
C11—C12—C13—C14	0.5 (4)	O1—Mn1—N3—N4	−83.4 (3)
C11—C12—C13—Br2	−178.1 (2)	N2—Mn1—N3—N4	106.2 (3)
C12—C13—C14—C15	−0.7 (4)	N1—Mn1—N3—N4	−171.8 (3)
Br2—C13—C14—C15	178.0 (2)	Mn1—N3—N4—N5	−177 (100)
C13—C14—C15—C16	−1.5 (4)	C2—C1—O1—Mn1	139.8 (2)
C14—C15—C16—O2	−174.8 (3)	C6—C1—O1—Mn1	−42.5 (3)
C14—C15—C16—C11	3.7 (4)	O2—Mn1—O1—C1	−138.02 (18)
C12—C11—C16—O2	174.7 (2)	N2—Mn1—O1—C1	112.3 (3)
C10—C11—C16—O2	−3.6 (4)	N1—Mn1—O1—C1	50.12 (18)
C12—C11—C16—C15	−3.8 (4)	N3—Mn1—O1—C1	−40.14 (19)
C10—C11—C16—C15	177.9 (2)	C15—C16—O2—Mn1	168.3 (2)
C6—C7—N1—C8	−177.5 (2)	C11—C16—O2—Mn1	−10.2 (4)
C6—C7—N1—Mn1	5.1 (4)	O1—Mn1—O2—C16	−144.9 (2)
C9—C8—N1—C7	−142.4 (3)	N2—Mn1—O2—C16	15.7 (2)
C9—C8—N1—Mn1	35.2 (3)	N1—Mn1—O2—C16	−30.2 (7)
O2—Mn1—N1—C7	−148.3 (5)	N3—Mn1—O2—C16	117.9 (2)
